# Multiple Cerebral Hematomas Revealing Infective Endocarditis: A Case Report and Literature Review

**DOI:** 10.7759/cureus.104208

**Published:** 2026-02-24

**Authors:** Mohamed Lamine Condé, Malé M Doré, Namory Camara, Mouloukou Souleymane Doumbouya, Fodé Abass Cissé

**Affiliations:** 1 Neurology, Ignace Deen University Hospital Center, Gamal Abdel Nasser University of Conakry, Conakry, GIN

**Keywords:** complication, conakry-guinea, infective endocarditis, multiple cerebral hematomas, simbaya

## Abstract

Multiple cerebral hematomas represent a significant neurological and neurosurgical emergency, posing both etiological and therapeutic challenges. The etiologies are numerous, including infective endocarditis. Intracerebral hemorrhage is an unusual complication of infective endocarditis and is rarely described as a mode of presentation, but it completely changes the prognosis and consequently the therapeutic approach.

Herein, we describe the case of a 32-year-old male patient with no history of valvular heart disease who presented to the emergency department with a febrile focal neurological deficit related to a hemorrhagic stroke and in whom diagnostic investigations identified infective endocarditis as the origin.

## Introduction

Infective endocarditis (IE) is a complex and fatal disease, combining cardiac involvement and multi-organ complications [1,2]. Cutaneous manifestations are present in only 5-25% of cases but are of great diagnostic value [3]. Neurological complications are present in less than 20% of IE, but they are often revealing and are a therapeutic emergency [4,5]. We present the observation of a 32-year-old male patient, with no history of heart disease or cardiovascular risk factors, who consulted at the Simbaya Conakry neurological clinic for a sudden onset of hemi-body motor deficit in the context of dermatosis and fever.

## Case presentation

A 32-year-old male patient presented with a sudden onset of left-sided hemiparesis and speech difficulties affecting articulation, accompanied by fever. He had a history of skin rash treated traditionally with decoctions (bath and oral administration). The onset of symptoms occurred a month prior to presentation, marked by palpitations with chest pain in the context of an unquantified fever. This motivated the patient to self-medicate with 1g of paracetamol and a combination of artemisinin without improvement, followed by left hemi-body motor deficit with joint disorder.

**Figure 1:**
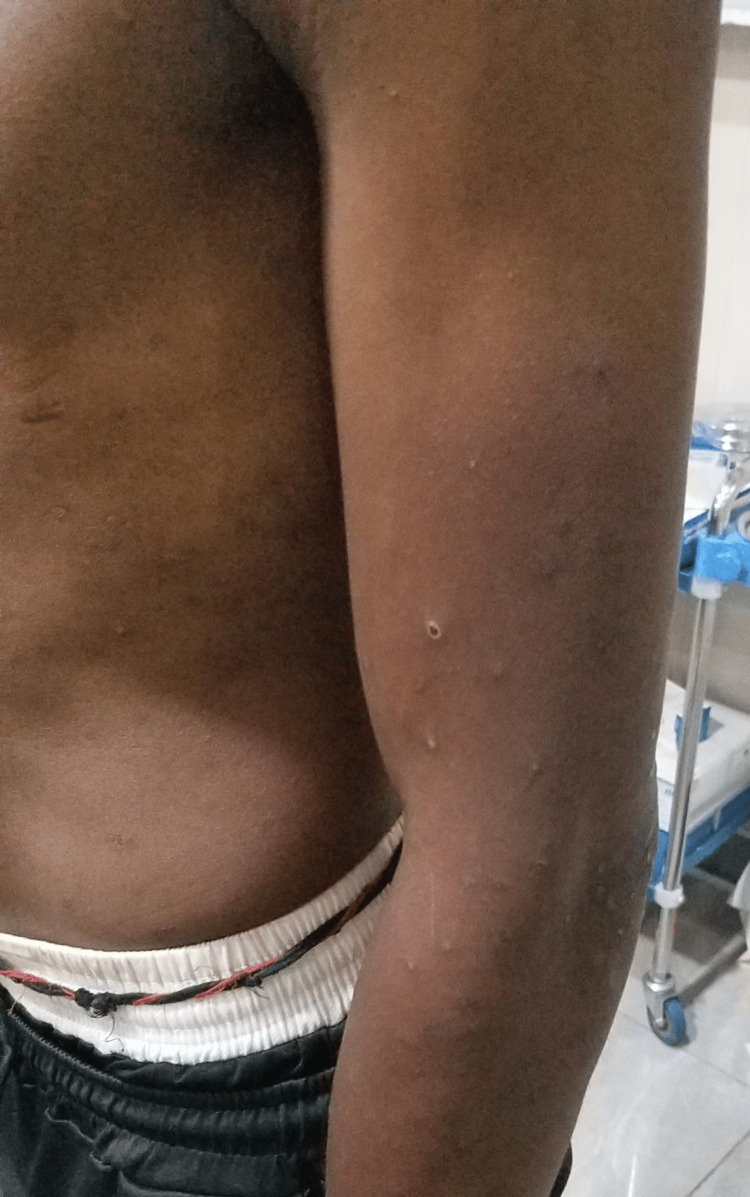
Folliculitis-like skin eruptions on the arm, forearm, and trunk

The clinical examination revealed a dysarthric patient with left hemiparesis rated 4/5 on the Medical Research Council scale (MRC) [6], brisk deep tendon reflexes, and a positive Babinski sign with a National Institutes of Health Stroke Scale (NIHSS) score of 4. Furthermore, folliculitis-like skin eruptions were noted along with a fever of 38.7°C (Figure 1). Hemodynamically, the patient was stable with a blood pressure of 130/80 mmHg and a heart rate of 109 bpm. He was eupneic with a respiratory rate of 20 cycles/min and a pulse oxygen saturation of 98% on room air.

**Figure 2:**
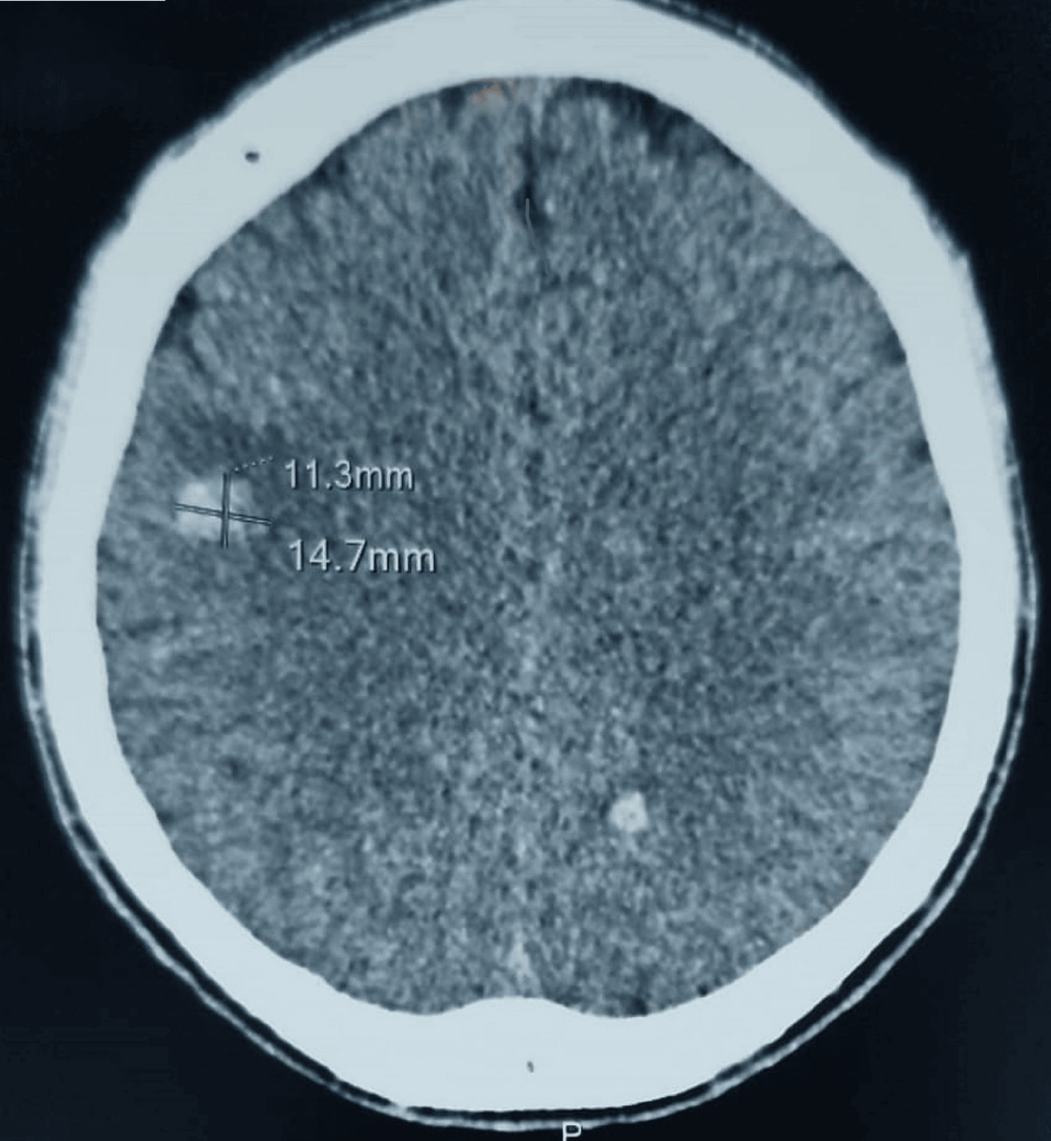
Brain CT scan Brain CT scan showing spontaneous hyperdensity in the right frontal and left parietal lobes without mass effect, suggestive of a multiple cerebral hematoma.

Given this neurological picture, we performed a brain scan without the injection of a contrast agent; this showed the presence of multiple bi-hemispheric intraparenchymal hematomas (Figure 2). The blood test showed white blood cells at 32,000 elements/mm^3^ with a predominance of neutrophils and a CRP of 267 mg/l (Table 1).

**Table 1:** Laboratory results MCV: mean corpuscular volume; MCH: mean corpuscular hemoglobin; MCHC: mean corpuscular hemoglobin concentration; MXD: mixed cells *Reference values are shown as men/women where applicable.

Analytics	Results	Units	Reference values
C-reactive protein	Positive (267)	mg/L	< 6
Leukocyte formula			
Leukocytes	32000	/mm^3^	4000 - 10000
Lymphocytes	855	/mm^3^	800 - 4000
Neutrophils	8500	/mm^3^	2000 - 7000
MXD	200	/mm^3^	100 - 1500
Erythrocyte formula			
Red blood cell	5.2	tera/liter	4 - 5.50/4.1 - 5.3*
Hemoglobin	13.0	g/dl	12 - 18/12 - 16*
Hematocrit	46.0	%	40 - 50/35 - 45*
MCV	84	fl	70 - 100
MCH	28.7	pg	27 - 32
MCHC	34.1	g/dl	31 - 35
Platelet count			
Blood platelets	300	giga/L	100 - 450

**Figure 3:**
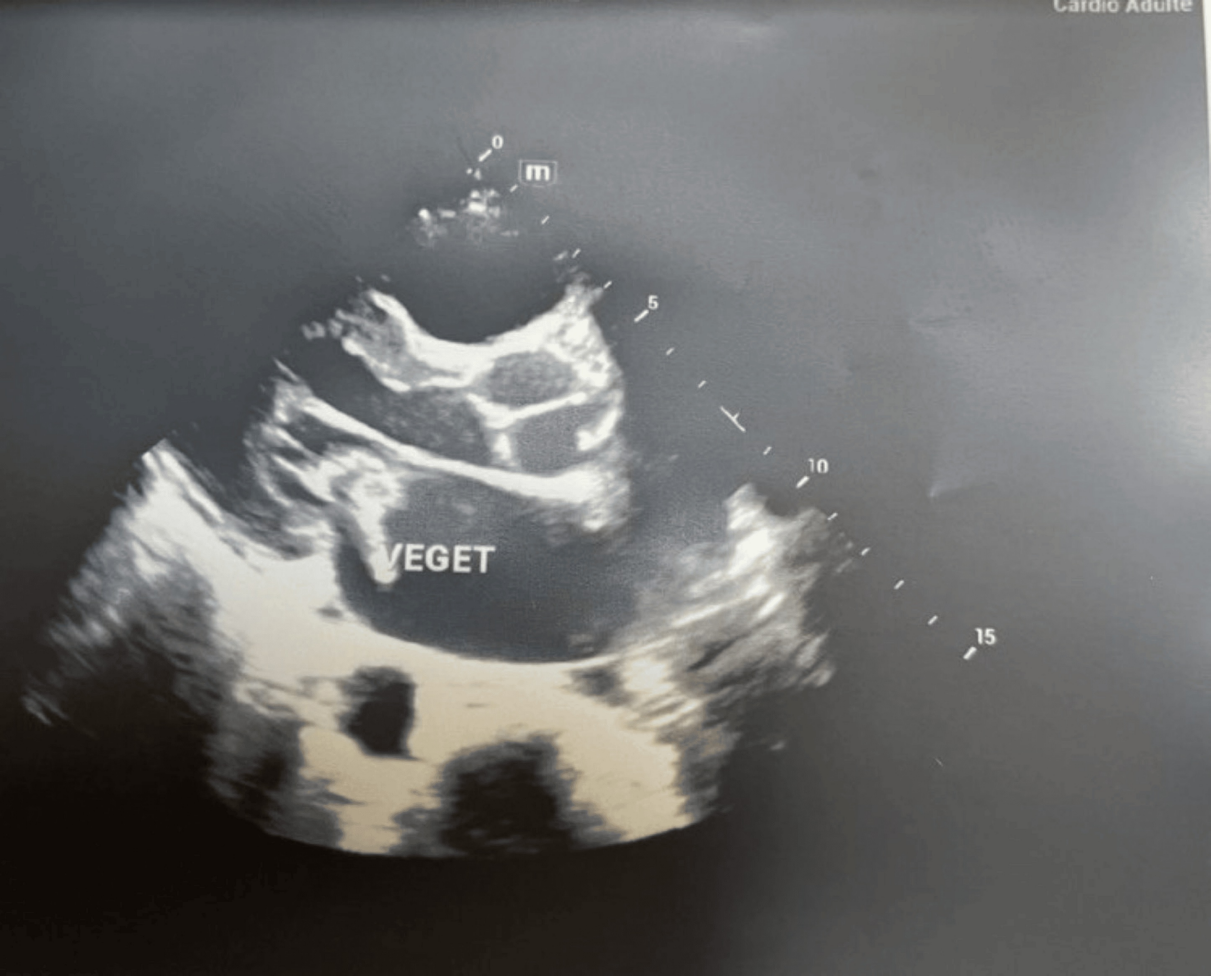
Transthoracic ultrasound image Transthoracic ultrasound image showing slightly thickened mitral valves, total restriction of the posterior valve, prolapse of A1, A2, and A3 associated with dilation of the mitral annulus responsible for severe eccentric leak, as well as the presence of a hyperechoic mass hanging on the atrial side of the anterior mitral valve, suspicious for vegetations.

Given the patient's normal blood pressure, skin rash, and fever, we performed a transthoracic echocardiogram to look for signs of infective endocarditis as part of the etiological workup for stroke. This revealed vegetation at the free edge of the large mitral valve with mitral insufficiency of rheumatic origin (Figure 3). Thus, a diagnosis of multiple intracerebral hemorrhage due to infective endocarditis was made, and the patient was placed on empirical antibiotic therapy combining ampicillin + oxacillin and gentamicin initially to cover gram-positive and gram-negative microorganisms with synergistic action before the results of the blood culture were available. The blood cultures identified methicillin-sensitive *Staphylococcus aureus *(*S. aureus*), and we readjusted antibiotic therapy to a combination of amoxicillin + clavulanic acid at a dose of 200mg/kg/day for four weeks. The evolution was marked by a stabilization of the infection with the achievement of apyrexia within 72 hours and a complete motor recovery by the third week.

## Discussion

Infective endocarditis is a rare condition with variable clinical manifestations and is generally difficult to diagnose. Its incidence is 1.5 cases per 100,000 per year. It can cause numerous systemic complications related either to immunological phenomena (immune complexes) or to the migration of septic emboli [7]. Neurological complications represent a significant category of complications, with their incidence varying between 10% and 35%, depending on the study. Their particularity lies in the fact that they affect the patient's life prognosis and therefore can completely change the management and/or the prognosis [7]. Intracranial hemorrhage accounts for 12-30% of neurological complications. It can affect either the subarachnoid spaces or the cerebral parenchyma. Three pathophysiological mechanisms have been identified: hemorrhagic transformation of an inferior vena cava (IVC); rupture of a vessel by necrotizing arteritis; rupture of an intracranial mycotic aneurysm [8].

Intracranial hemorrhage as a manifestation of IE is rare in the pediatric population and accounts for less than 20% of IE cases [9,5]. The vast majority of neurological complications most often occur during the course of an already diagnosed IE, or during the assessment of the extent of asymptomatic forms [5]. To our knowledge, our patient's case is one of the rare reported cases of this type of presentation in the adult population. This could be explained by inadequate treatment of the dermatosis (treated traditionally with decoction baths and oral administration) on the one hand, and by diagnostic delay on the other.

Brain imaging (CT scan, MRI) is essential for the diagnosis of neurological complications of IE [10]. The lesions are most often multiple, variously combining ischemic images, hemorrhages, abscesses, or mycotic aneurysms [11,12]. These complications can be the initial ones, as in the case of our observation, where cerebral computed tomography without injection of contrast medium showed two hemispheric intracerebral hematomas.

IE always follows bacteremia, with the entry point being cutaneous in 20% of cases [3]. The specific adhesion capabilities of each microorganism, as well as the extent and duration of bacteremia, are determining factors. The normal skin bacterial flora consists of 63% coagulase-negative staphylococci and 30% *S. aureus* [13]. Chronic, extensive dermatoses that are partially controlled or even refractory to treatment are a gateway that should not be underestimated [3]. In our patient, we found lesions of atopic dermatitis that he traditionally treated with decoctions for bathing and drinking; blood cultures identified *S. aureus* as the causative microorganism. In a study by Konstantinou et al., three patients with IE to *S. aureus* on native valve had been initially admitted for flares of chronic dermatoses, treated in all cases with topical or systemic immunosuppressants [3].

## Conclusions

The diagnosis and treatment of infective endocarditis remain challenging and particularly difficult. It should be considered when there is a combination of fever, neurological deficits, and skin signs, with evidence of vegetation on echocardiography and positive blood cultures for* S. aureus*.

Poorly treated chronic skin diseases are a gateway that should not be underestimated. Early diagnosis allows for the initiation of appropriate antibiotic therapy and improved prognosis.
